# Using Machine Learning Algorithms to Predict People’s Intention to Use Mobile Learning Platforms During the COVID-19 Pandemic: Machine Learning Approach

**DOI:** 10.2196/24032

**Published:** 2021-02-04

**Authors:** Iman Akour, Muhammad Alshurideh, Barween Al Kurdi, Amel Al Ali, Said Salloum

**Affiliations:** 1 Information Systems Department University of Sharjah Sharjah United Arab Emirates; 2 Department of Management University of Sharjah Sharjah United Arab Emirates; 3 Department of Marketing The University of Jordan Amman Jordan; 4 Department of Business Administration Faculty of Economics and Administrative Sciences The Hashemite University Zarqa Jordan; 5 Research Institute of Sciences & Engineering University of Sharjah Sharjah United Arab Emirates

**Keywords:** COVID-19, pandemic, mobile learning, fear, technology acceptance model, theory of planned behavior, prediction, intent, online learning, machine learning, behavior

## Abstract

**Background:**

Mobile learning has become an essential instruction platform in many schools, colleges, universities, and various other educational institutions across the globe, as a result of the COVID-19 pandemic crisis. The resulting severe, pandemic-related circumstances have disrupted physical and face-to-face contact teaching practices, thereby requiring many students to actively use mobile technologies for learning. Mobile learning technologies offer viable web-based teaching and learning platforms that are accessible to teachers and learners worldwide.

**Objective:**

This study investigated the use of mobile learning platforms for instruction purposes in United Arab Emirates higher education institutions.

**Methods:**

An extended technology acceptance model and theory of planned behavior model were proposed to analyze university students’ adoption of mobile learning platforms for accessing course materials, searching the web for information related to their disciplines, sharing knowledge, and submitting assignments during the COVID-19 pandemic. We collected a total of 1880 questionnaires from different universities in the United Arab Emirates. Partial least squares-structural equation modeling and machine learning algorithms were used to assess the research model, which was based on the data gathered from a student survey.

**Results:**

Based on our results, each hypothesized relationship within the research model was supported by our data analysis results. It should also be noted that the J48 classifier (89.37% accuracy) typically performed better than the other classifiers when it came to the prediction of the dependent variable.

**Conclusions:**

Our study revealed that teaching and learning could considerably benefit from adopting remote learning systems as educational tools during the COVID-19 pandemic. However, the value of such systems could be lessened because of the emotions that students experience, including a fear of poor grades, stress resulting from family circumstances, and sadness resulting from a loss of friends. Accordingly, these issues can only be resolved by evaluating the emotions of students during the pandemic.

## Introduction

### Background

Colleges and universities often actively aim to create web-based teaching environments with the help of relevant learning platforms and resources [1-3]. In addition, these higher education institutions attempt to achieve effective student results by providing various learning management platforms that enhance strategies and practices for teaching and learning. However, the COVID-19 pandemic has presented higher education institutions with several challenges, as students worldwide have been experiencing negative emotions and feelings with regard to their studies. Such emotions include fear, anxiety, and apprehension. A consequence of these negative emotions is stigmatization, which students who are mentally affected by fear often experience. In addition, students have experienced discrimination, loss, and various other psychosocial issues after COVID-19 was declared a pandemic [4-6]. The lockdown effect has also had an impact on students’ fear; the need for e-learning became critical when education institutes were forced to halt their contact learning and teaching practices. Furthermore, students’ fear can manifest as a fear of taking risks, a fear of failure, a fear of missing out, and fear resulting from insecurity [7-10]. Students’ fear can also impact technology adoption, as the COVID-19 lockdown has forced universities, colleges, and schools to implement distance learning in an attempt to lessen the harmful effects of COVID-19 and maintain student learning.

A considerable percentage of colleges and universities have experienced issues that relate to educators’ experience with using technology for teaching and learning. The technological proficiency of students is also problematic, as classes need to be conducted via web-based methods [11-15]. However, adopting technology for distance learning is essential for efficiently validating the conduction of web-based classes [16-19]. According to the majority of technology adoption studies, there are complications with regard to the adoption process, as technology adoption can affect other teaching and learning factors, such as learning strategies, learning contexts, and technology availability.

Although several researchers have focused on technology adoption in their research, the adoption of creative teaching methods (eg, the use of mobile learning apps) as a result of the COVID-19 pandemic and other similar disasters has yet to be explored. It has become quite easy to find mobile learning apps on both the Apple Store and Google Play Store. Users can access mobile learning apps from these stores, which are responsible for automatically updating these apps. In addition, users have been increasingly accessing these apps because of app stores’ freemium approach [20,21]. However, students’ and educators’ thoughts on implementing a mobile learning platform during the pandemic must be considered. Therefore, the need for mobile learning platforms and the issues surrounding the COVID-19 pandemic need to be addressed [22]. As the use of mobile learning platforms is a relatively new practice, there is a lack of research on how mobile learning can influence higher education. Furthermore, although the technology adoption domain has undergone extensive research, there has been a lack of focus on the emotion of fear when considering the adoption of technology during the COVID-19 pandemic. Past studies have mostly dealt with the technological factors in teaching and learning, without paying any attention to psychological factors. The impact of fear on technology adoption has yet to be clearly understood, and this is often the reason why technology has not been used to its full potential when it comes to the education domain [23].

After taking into consideration the limitations of technology adoption in education, we aimed to provide educational information on appropriate technology use, for times when learners and educators are fearful of technology. This is particularly relevant at times (eg, the COVID-19 pandemic) when technology use becomes imperative for providing better education to both learners and educators, who are often novices in terms of using technological applications for teaching and learning.

When it comes to the academic research adoption model, studies have found that using the technology acceptance model (TAM) and the theory of planned behavior (TPB) model as a hybrid model is effective for technology adoption. With the help of these models, it becomes possible to determine users’ willingness to accept and use technology [24,25]. Accordingly, this study focuses on understanding students’ and educators’ willingness to use mobile learning systems, by using the TPB model and TAM, in addition to 2 external factors (ie, subjective norms [SNs] and fear). As a result, we were able to use the TAM and TPB model to investigate students’ and teachers’ thoughts on using machine learning methods during the spread of COVID-19. In addition, assessments of fear during the COVID-19 pandemic and how fear directly affects the TAM and TPB model have been limited. After considering the lack of research, we aimed to develop a hybrid model that can determine the different fears that both learners and educators may face during the COVID-19 pandemic. Since we investigated the factor of fear, we believe that our research paper has an increased chance of providing both teachers and app developers with the technology and education-related information needed for developing and implementing new technologies during the COVID-19 lockdown period.

The unique educational problems that have emerged during these unordinary times can be highlighted if more information on the factors of machine learning adoption at the time of the COVID-19 pandemic is gathered. COVID-19–related literature on the technology adoption domain can benefit higher education institutions on a theoretical and practical level.

### Literature Review

Previous research studies on technology adoption have focused on the various forms of fear [23,26]. For example, anxiety is an important factor that helps manage technology approval and apprehension. Within the education sector, the adoption of technology by students is influenced by anxiety [27]. Furthermore, apart from anxiety, a lack of experience and skills may also influence technology use. The fear of using technology, combined with poor technological literacy and anxiety, negatively affects the adoption of technology. Hence, it is essential for teachers and educators to focus on psychological development and help students accept the use of technology. Other factors of the fear of using technology within the educational sector include technical readiness and preparedness; technology adoption is negatively influenced by both of these factors [28-30].

The education sector is not the only sector that has exhibited a fear of technology adoption. Medical sector students usually perceive risks and exhibit negative anxiety when technology is used [31,32]. In addition, health anxiety is one of the top concerns of the health care sector. Health anxiety includes the apprehension of patients and the fear of receiving results about a severe illness. With regard to the banking sector, various kinds of fear that relate to customers’ perceptions and attitudes toward technology have been recognized. Customers do not want to use their data for mobile payments. Customers fear the use of technology in mobile banking and are negatively influenced by the frauds that have occurred. As a result, they lack both technological experience and trust in technology [33,34]. With regard to the household sector, the main reasons why technology is not being used include the fear of using technology and the fear that technology will increase the number of family tasks [23].

Various research studies have assessed the issues that relate to technological acceptance and fear. These research studies are based on the TAM [29,30,32-35] and several other models [28,31,36,37], and most of these research studies have assessed how the fear of technology can influence technology acceptance. Various technology users have provided justifications for their fear of technology use. For example, several users have stated that their fear is related to self-confidence. Errors are made when a human is assigned to a job, and excessively worrying about this fact enhances fear [38]. Moreover, several users have stated that they do not use technology because they believe that technology is time-consuming, and therefore does not allow them to complete their tasks [39]. Various technology acceptance studies have assessed the influence of fear on the breach of data privacy, and this is why privacy and security awareness are emphasized in technology research studies [40].

Previous studies have not provided sufficient empirical research on the use of mobile learning in United Arab Emirates (UAE) institutions, nor have they considered the factors that influence students’ actual technology use. When it comes to methodology, technology acceptance researchers have typically analyzed theoretical models by using structural equation modeling and machine learning algorithms. After considering various theoretical models, we conducted this study with the following 2 objectives: (1) examine how students use mobile learning by integrating the TAM [41] and TPB model [42] into 1 theoretical model, and (2) validate the created theoretical model with the help of machine learning and partial least squares-structural equation modeling (PLS-SEM) algorithms.

### Theoretical Model and Research Model

#### Model Design

**Figure 1:**
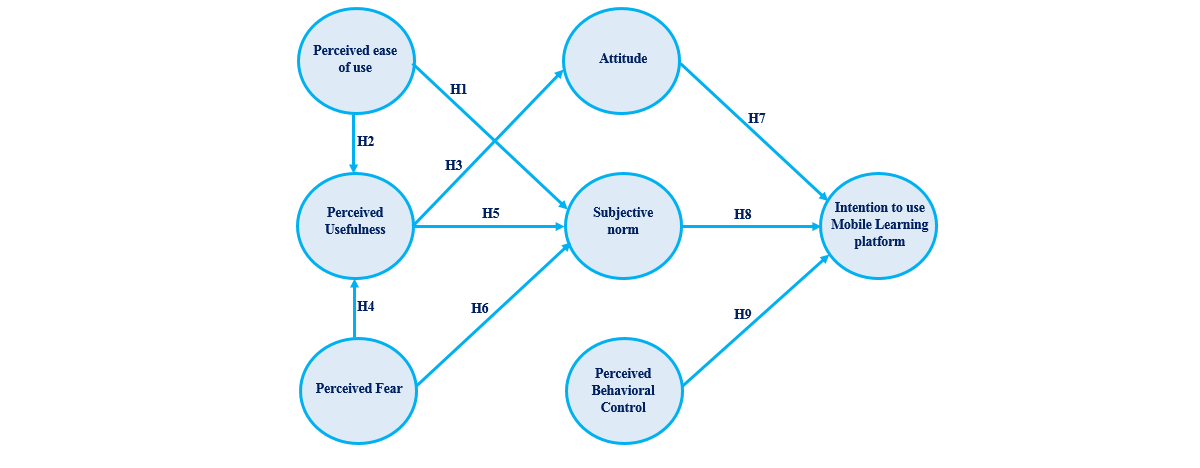
Study model.

In this study, the research model was developed to integrate the SN and fear constructs into 2 kinds of theoretical models—the TAM and TPB model. We believed that the SN and fear would influence the perceived ease of use (PEOU) and perceived usefulness (PU) of mobile learning systems. Additionally, we believed that attitude and perceived behavioral control (PBC) would be influenced by the continuous intention to use mobile learning systems. The proposed theoretical model is presented in Figure 1.

#### TAM

One of the main objectives of the TAM is to validate external factors based on personal belief. The model is considered quite powerful, since it can be used to explain individuals’ ability to accept the technology at their educational institutions [41,43-45]. According to the TAM, the 2 kinds of perceptions that can be measured are PU and the PEOU. This means that the behavioral intention of the user can be influenced directly. PU should be considered because this factor helps with measuring the degree to which technology must be evaluated by an individual, and assessing whether a technology is useful enough to be adopted and accepted. However, the PEOU refers to the degree to which an individual believes that technology is manageable and attainable [41].

In the context of technology acceptance, attitude has been defined as a user’s desire to use a system [46]. Previous mobile learning studies have indicated that behavioral intention and attitude are related to each other. Previous research has also suggested that the intention to use mobile learning systems is significantly influenced by attitude [47-50].

Keeping in mind the previous assumptions, it can be concluded that if technology is considered to be easy to use, then users will retain a positive attitude. Therefore, user perceptions are quite important. If users have a positive attitude, it is believed that the users will adopt technology. The following hypotheses were proposed after applying the previous assumptions to the research model: (1) the PEOU will predict the SN (ie, H1), (2) the PEOU will predict PU (ie, H2); (3) PU will predict attitude (ie, H3), (4) PU will predict the SN (ie, H5), and (5) people’s attitudes will predict their intention to use a mobile learning platform (ie, H7).

#### SN

Individual perceptions can be measured by using a tool called the SN, which is a type of perception that is based on the presence of individuals who exhibit similar attitudes and behaviors toward technology. The TAM is strengthened by the SN, since the TAM has been enabled to integrate user behaviors that are present within a user group [51]. The SN is an external factor that includes students’ intentions to adopt mobile learning technology for classmate group meetings.

Various literature on technology adoption or acceptance have shown that the SN also influences behavioral intention, PU, and the PEOU [45,52-54]. The SN and TAM have recently been used as external factors in a study by Huang et al [55], who stated that the TAM-embedded factors from various research studies had a significantly close relationship with external factors. However, they found that the external factor SN was not efficiently or deeply implemented in other studies. Previous studies have stated that the intention of using mobile learning platforms is significantly influenced by the SN [49,50,56-58]. Hence, the following hypothesis was developed: the SN will predict people’s intention to use a mobile learning platform (ie, H8).

#### Perceived Fear

On December 2019, the novel COVID-19 disease was observed in China, and with time, it spread throughout the world. Based on recent studies, the reaction toward the perceived threat of the SARS-CoV-2 virus has been fear. Additionally, the Health Anxiety Inventory scale has shown that fear is at the highest level [59]. Even though fear is perceived to be positive when real dangers are present, fear in the context of the COVID-19 pandemic may be burdensome and chronic. There are various forms of fear that are related to the COVID-19 pandemic, including health anxiety, uncertainty, and the fear of the risk of losing loved ones. The COVID-19 pandemic has resulted in the development of 2 vital issues, as follows: a high degree of worrying and a high possibility of being affected by the disease [4,60].

This study aimed to analyze the association between the adoption of technology and the external factor perceived fear (PF), through the use of the TAM. In this study, TAM limitations needed to be overcome. Such limitations include the implementation of external factors that are specific to the analysis of a TAM for PF, including PU, the PEOU, and the SN [61]. Hence, the following hypotheses were developed while keeping these factors in mind: PF will predict PU (ie, H4), and PF will predict the SN (ie, H6).

#### PBC

PBC is defined as “people’s perception of the ease or difficulty of performing the behavior of interest” [62]. Previous research has shown that the intention to use mobile learning platforms is significantly affected by PBC [49,50,63]. Hence, the following hypothesis was proposed: PBC will predict people’s intention to use mobile learning platforms (ie, H9).

Our hypotheses were used to develop the proposed research model, as indicated in Figure 1. The theoretical model was presented as a structural equation model and analyzed with machine learning methods.

## Methods

### Context and Subjects

University students were the target population for this study. The questionnaire was disseminated to university students in the UAE. In total, 7 well-known universities in the UAE were chosen for this study, namely the University of Sharjah, the Higher Colleges of Technology, The British University in Dubai, United Arab Emirates University, the University of Fujairah, American University in UAE, and Ajman University. We used a web-based survey to collect data from May to June 2020. The surveys were completed by the participants, who did not ask for any compensation. In this study, the convenience sampling technique was used for data collection. In total, 2000 surveys were distributed, and a 94% response rate was recorded (ie, 1880 students completed the whole survey). The number of males and females who completed the survey was 1102 (58.6%) and 778 (41.4%), respectively.

The percentage of participants aged 18-29 was 40.3% (758/1880), and the remaining 59.7% of participants (1122/1880) were older than 29 years. Furthermore, 33.3% (626/1880) of the participants were undergraduate students, 45.2% (849/1990) were master students, 11.1% (209/1880) were PhD students, and 10.4% (196/1880) were diploma students. A comprehensive view of the collected data is provided in Tables 1 and 2.

**Table 1:** Number of students (N=1880) in participating universities.

University	Number of students, n
United Arab Emirates University	568
University of Sharjah	439
Higher Colleges of Technology	365
Ajman University	287
The British University in Dubai	103
University of Fujairah	68
American University in United Arab Emirates	50

**Table 2:** Summary of students’ demographic characteristics.

Variables		Participants, n (%)
**Gender**		
	Male	1102 (58.6)
	Female	778 (41.4)
**Age (years)**		
	18-29	758 (40.3)
	30-39	635 (33.7)
	40-49	367 (19.5)
	50-59	120 (6.5)
**Level of education**		
	Diploma	196 (10.4)
	Bachelor degree	626 (33.3)
	Master degree	849 (45.2)
	PhD degree	209 (11.1)

### Study Design

This study’s design consisted of 2 parts. The first part focused on collecting participants’ demographic data. The second part focused on collecting responses that were related to the factors in the conceptual model’s 5-point Likert scale. To assess the 7 constructs (ie, attitude, intention to use a mobile learning platform, SN, PBC, PF, PEOU, and PU) in the questionnaire, 20 items were included in the survey. The sources of these constructs are presented in Table 3.

**Table 3:** Constructs and their sources.

Construct	Number of items, n	Source, authors
Attitude	3	Al-Emran et al [49], Cheon et al [50]
Intention to use a mobile learning platform	2	Al-Emran et al [49], Tan et al [64], Bao et al [65]
Subjective norm	3	Al-Emran et al [49], Cheon et al [50]
Perceived behavioral control	3	Al-Emran et al [49], Cheon et al [50]
Perceived fear	3	Developed in this study.
Perceived ease of use	3	Al-Emran et al [49], Tan et al [64], Bao et al [65]
Perceived usefulness	3	Al-Emran et al [49], Tan et al [64], Bao et al [65]

### Questionnaire Pretest

Before conducting the final survey, it was important to make sure that the questionnaire items were reliable by conducting a pilot study with a random selection of 100 students from the target population. We calculated Cronbach α values to measure the internal reliability of the items of each construct. Nunnaly and Bernstein [66] have suggested that an acceptable reliability coefficient should equal at least .70. Table 4 shows that this study’s constructs had Cronbach α values of >.70. Therefore, each construct was reliable. This meant that each construct could be used in the final research model.

Table 3 shows that the questionnaire's 5-point Likert scales were reliable. Therefore, the measurement scales could be used in this study.

**Table 4:** Cronbach α values for the pilot study (Cronbach α≥.70).

Construct	Cronbach α
Attitude	.736
Intention to use a mobile learning platform	.755
Subjective norm	.864
Perceived behavioral control	.859
Perceived fear	.847
Perceived ease of use	.887
Perceived usefulness	.803

## Results

### Data Analysis

The theoretical model developed in this study was evaluated by using 2 different techniques. The first technique involved PLS-SEM and the use of the SmartPLS (SmartPLS GmbH) tool [67]. This study used the PLS-SEM technique, mainly because both the structural and measurement models could be concurrently analyzed through PLS-SEM, thereby increasing the preciseness of results [68]. As for the second technique, we predicted the dependent variables of the conceptual model with the help of machine learning algorithms in Weka (University of Waikato) [69].

### Model Reliability and Validity Assessment

We assessed the validity and reliability of the measurement model [70]. Model reliability was tested by using Cronbach α and composite reliability measures. It has been suggested that these measures must equal at least .70 to be acceptable [70]. As per the results in Table 5, model reliability was confirmed, as satisfactory values were attained for both measures.

According to Hair Jr et al [70], discriminant and convergent validities can be evaluated to test model validity. We calculated the factor loading and average variance extracted values of each construct item to determine convergent validity. It has been suggested that the average variance extracted and factor loading values must equal at least .50 [71] and .70 [72], respectively, to be acceptable. As per the results in Table 5, convergent validity was confirmed, as accepted values were attained for both measures. Furthermore, Henseler et al [73] have suggested that the Heterotrait-Monotrait ratio of correlations can be calculated to determine discriminant validity. Heterotrait-Monotrait ratio values must fall below .85 to be acceptable. As per the results in Table 6, discriminant validity was confirmed, as accepted Heterotrait-Monotrait ratio values were attained.

**Table 5:** Convergent validity test results. Acceptable values (ie, factor loading, Cronbach α, CR^a^≥0.70, and AVE^b^>0.5) were obtained.

Constructs and items		Factor loading	Cronbach α	CR	AVE
**Attitude**			.798	.823	.760
	ATT1	.726			
	ATT2	.886			
	ATT2	.800			
**Intention to use a mobile learning platform**			.739	.789	.703
	INT1	.846			
	INT2	.805			
**Subjective norm**			.758	.811	.716
	SN1	.819			
	SN2	.795			
	SN3	.883			
**Perceived behavioral control**			.843	.771	.652
	PBC1	.822			
	PBC2	.873			
	PBC3	.778			
**Perceived fear**			.779	.798	.593
	PF1	.808			
	PF2	.845			
	PF3	.866			
**Perceived ease of use**			.769	.746	.633
	PEOU1	.872			
	PEOU2	.832			
	PEOU3	.857			
**Perceived usefulness**			.715	.750	.785
	PU1	.878			
	PU2	.906			
	PU3	.848			

^a^CR: composite reliability.

^b^AVE: average variance extracted.

**Table 6:** HTMT^a^ ratios of correlations between each construct.

Construct	Attitude	Intention to use a mobile learning platform	Subjective norm	Perceived behavioral control	Perceived fear	Perceived ease of use	Perceived usefulness
Attitude, HTMT ratio	**—** ^b^	.480	.519	.377	.330	.549	.651
Intention to use a mobile learning platform, HTMT ratio	.480	**—**	.299	.583	.514	.350	.504
Subjective norm, HTMT ratio	.519	.299	**—**	.516	.460	.393	.511
Perceived behavioral control, HTMT ratio	.377	.583	.516	**—**	.602	.657	.542
Perceived fear, HTMT ratio	.330	.514	.460	.602	**—**	.263	.494
Perceived ease of use, HTMT ratio	.549	.350	.393	.657	.263	**—**	.333
Perceived usefulness, HTMT ratio	.651	.504	.511	.542	.494	.333	**—**

^a^HTMT: Heterotrait-Monotrait ratio.

^b^Not applicable.

### Hypotheses Testing and Coefficient of Determination

**Figure 2:**
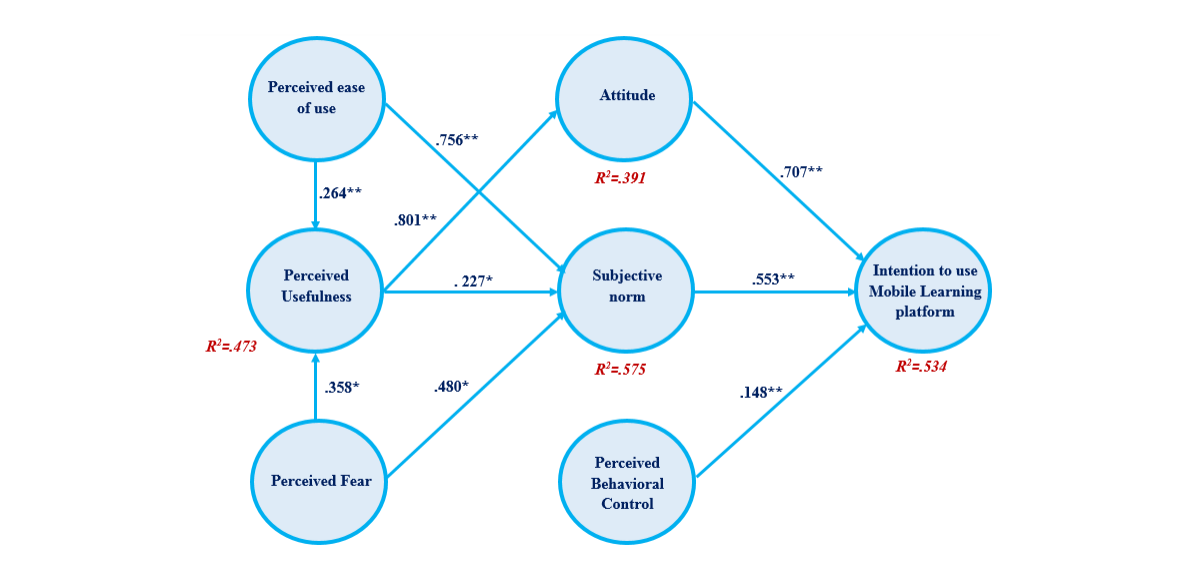
Hypotheses testing results. The R^2^ values reported are for perceived usefulness, attitude, the subjective norm, and the intention to use a mobile learning platform. The β values and statistical significance of each path are also reported. *significant at *P*<.05, **significant at *P*≤.01.

The 9 hypotheses we proposed were tested by using the structural equation modeling procedure [74]. Analyses were carried out to determine the variance (ie, the R^2^ value) of each path, the variance of the research model, and the significance of each hypothesized path association. Figure 2 and Table 7 show the standardized path coefficients and path significances.

The R^2^ values for attitude, intention to use a mobile learning platform, the SN, and PU ranged between 0.391 and 0.575, as shown in Table 7. Hence, these constructs had a moderate predictive power [75]. Based on the hypothesis data analysis, the empirical data supported every hypothesis (ie, H1, H2, H3, H4, H5, H6, H7, H8, and H9).

Table 8 and Figure 2 summarize the results of the hypotheses tests, which indicated that the SN significantly influenced the PEOU (β=.756; *P*=.001), PU (β=.227; *P*=.03) and PF (β=.480; *P*=.04). These results supported hypotheses H1, H5, and H6, respectively. PU had significant effects on attitude (β=.801; *P*<.001), which supports hypothesis H3. The results also revealed that the intention to use a mobile learning platform significantly influenced attitude (β=.707; *P*<.001), the SN (β=.553, *P*<.001), and PBC (β=.148, *P*<.001). These results supported hypotheses H7, H8, and H9, respectively. Additionally, the results show that PU was significantly influenced by the PEOU (β=.264; *P*=.002) and PF (β=.358; *P*=.04). These results supported hypotheses H2 and H4, respectively.

**Table 7:** R^2^ values of the endogenous latent variables.

Constructs	R^2^	Predictive power
Perceived usefulness	0.473	Moderate
Attitude	0.391	Moderate
Subjective norm	0.575	Moderate
Intention to use a mobile learning platform	0.534	Moderate

**Table 8:** Summary of hypotheses testing results.

Hypothesis	Relationship	Path β	*t* test (df)^a^	*P* value	Correlation direction	Decision
H1	Perceived ease of use and subjective norm	.756	18.179 (1876)	.001	Positive	Supported^b^
H2	Perceived ease of use and perceived usefulness	.264	10.203 (1876)	.002	Positive	Supported^c^
H3	Perceived usefulness and attitude	.801	19.093 (1876)	<.001	Positive	Supported^b^
H4	Perceived fear and perceived usefulness	.358	4.936 (1876)	.04	Positive	Supported^d^
H5	Perceived usefulness and subjective norm	.227	4.660 (1876)	.03	Positive	Supported^d^
H6	Perceived fear and subjective norm	.480	5.892 (1876)	.04	Positive	Supported^d^
H7	Attitude and intention to use a mobile platform	.707	15.337 (1876)	<.001	Positive	Supported^b^
H8	Subjective norm and intention to use a mobile platform	.553	19.485 (1876)	<.001	Positive	Supported^b^
H9	Perceived behavioral control and intention to use a mobile platform	.148	18.089 (1876)	<.001	Positive	Supported^b^

^a^The *t* test conducted was 2-tailed.

^b^The hypothesis is supported based on a significant *P* value of ≤.001.

^c^The hypothesis is supported based on a significant *P* value of ≤.01.

^d^The hypothesis is supported based on a significant *P* value of <.05.

### Hypotheses Testing With Machine Learning Algorithms

This study was conducted with the assistance machine learning classification algorithms, which were applied through various methodologies, such as neural networks, if-then-else statements, decision trees, and Bayesian networks. Machine learning algorithms were used to predict the relationships in the proposed theoretical model [69,76,77]. With the help of Weka (version 3.8.3), the predictive model was tested on the basis of different classifiers, such as the OneR, J48, Logistic, LWL (Locally Weighted Learning), AdaBoostM1, and BayesNet classifiers [78,79]. In terms of predicting the PU of mobile learning systems, the J48 classifier performed better than the other classifiers, as seen from the results in Table 9. In the 10-fold cross-validation, the J48 classifier had an accuracy of 83.76% when predicting PU. Accordingly, these results supported hypotheses H2 and H4. The J48 classifier performed better than the other classifiers because of its high true positive rate (.837), precision (.803) and recall value (.838).

In terms of predicting attitude, the J48 classifier performed better than the other classifiers, as seen from the results in Table 10. The J48 classifier was able to use PU to predict attitude with an accuracy of 80.13%. Accordingly, these results supported hypothesis H3.

The results in Table 11 suggest that the J48 classifier performed better than the other classifiers when it came to predicting the SN based on the PEOU, PU, and PF. By using these constructs, the J48 classifier could predict the SN with an accuracy of 89.37%. Accordingly, these results supported hypotheses H1, H5, and H6.

According to the results in Table 12, the J48 classifier performed better than the other classifiers when it came to predicting the intention to use a mobile learning platform based on attitude, the SN, and PBC. When predicting the intention to use a mobile learning platform, the J48 classifier had an accuracy of 86.66%. These results supported hypotheses H7, H8, and H9.

**Table 9:** Predicting perceived usefulness based on the perceived ease of use and perceived fear.

Classifier	CCI^a^, %	TP^b^ rate	FP^c^ rate	Precision	Recall	F measure
BayesNet	80.11	.801	.295	.721	.801	.790
Logistic	81.02	.810	.308	.735	.810	.798
LWL^d^	80.54	.805	.339	.705	.810	.801
AdaBoostM1	82.10	.821	.338	.732	.821	.819
OneR	81.66	.816	.337	.712	.820	.816
J48	83.76	.837	.634	.803	.838	.828

^a^CCI: correctly classified instances.

^b^TP: true positive.

^c^FP: false positive.

^d^LWL: Locally Weighted Learning.

**Table 10:** Predicting attitude based on perceived usefulness.

Classifier	CCI^a^, %	TP^b^ rate	FP^c^ rate	Precision	Recall	F measure
BayesNet	78.02	.780	.229	.735	.781	.726
Logistic	77.22	.772	.205	.737	.723	.728
LWL^d^	76.79	.767	.269	.700	.768	.687
AdaBoostM1	78.11	.781	.289	.745	.782	.776
OneR	79.61	.796	.301	.754	.800	.798
J48	80.13	.801	.480	.787	.801	.800

^a^CCI: correctly classified instances.

^b^TP: true positive.

^c^FP: false positive.

^d^LWL: Locally Weighted Learning.

**Table 11:** Predicting the subjective norm based on the perceived ease of use, perceived usefulness, and perceived fear.

Classifier	CCI^a^, %	TP^b^ rate	FP^c^ rate	Precision	Recall	F measure
BayesNet	80.76	.807	.311	.760	.810	.758
Logistic	80.63	.806	.369	.762	.810	.759
LWL^d^	80.06	.800	.299	.756	.801	.748
AdaBoostM1	81.37	.813	.378	.763	.814	.760
OneR	82.79	.827	.409	.772	.833	.772
J48	89.37	.893	.598	.788	.894	.782

^a^CCI: correctly classified instances.

^b^TP: true positive.

^c^FP: false positive.

^d^LWL: Locally Weighted Learning.

**Table 12:** Predicting the intention to use a mobile learning platform based on attitude, the subjective norm, and perceived behavioral control.

Classifier	CCI^a^, %	TP^b^ rate	FP^c^ rate	Precision	Recall	F measure
BayesNet	81.10	.811	.303	.753	.812	.750
Logistic	81.23	.812	.371	.758	.813	.752
LWL^d^	80.73	.807	.389	.751	.812	.750
AdaBoostM1	81.44	.814	.369	.762	.815	.761
OneR	83.76	.837	.396	.770	.841	.768
J48	86.66	.866	.595	.802	.872	.798

^a^CCI: correctly classified instances.

^b^TP: true positive.

^c^FP: false positive.

^d^LWL: Locally Weighted Learning.

## Discussion

### Principal Findings

To test our proposed model, we used a complementary approach that combined the use of PLS-SEM and machine learning classification algorithms. There are few studies that have aimed to use machine learning algorithms to predict the actual use of mobile learning systems. Accordingly, studies that use a complementary multianalytical approach can play a major role in information systems literature and research. It should also be noted that PLS-SEM can help with predicting a dependent variable and validating a conceptual model that aims to extend an existing theory [80]. Similarly, a dependent variable can also be predicted with the help of supervised machine learning algorithms (ie, machine learning algorithms with a predefined dependent variable) and independent variables [69]. Another aspect of our study was the use of various classification algorithms in conjunction with the application of various methodologies, including if-then-else rules, neural networks, association rules, Bayesian networks, and decision trees. The J48 decision tree typically performed better than the other classifiers, as determined by our findings. Furthermore, we used a nonparametric decision tree to classify both categorical and continuous (ie, numerical) variables to obtain homogeneous subsamples from our main sample, on the basis of the main independent variable [69]. In other words, we used the nonparametric PLS-SEM technique to determine the significance of coefficients by using sample replacements, which were drawn from numerous subsamples on a random basis. This analysis provided empirical evidence for the impact of using mobile learning platforms during the COVID-19 pandemic. Our hypotheses (ie, H1, H5 and H6) significantly and positively supported the relationships between the SN and PEOU (*P*=.001), the SN and PU (*P*=.03), and the SN and PF (*P*=.04). Numerous research studies have assessed the relationship between the SN and PEOU, the SN and PU, and the SN and PF [23,41,43-45]. Moreover, our analysis provided empirical evidence for the effect of the PEOU on PU, as proposed in hypothesis H2. Our results showed that this effect was positive and significant (*P*=.002). Therefore, hypothesis H2 was in line with the findings of various studies [47-50].

Our analysis also provided empirical evidence for the effect of PU on attitude, as proposed in hypothesis H3. Our results showed that the effect was positive and significant (*P*<.001). Therefore, hypothesis H3 was in line with the findings of various studies [41,43-45,49]. PF also had a significant effect on PU (*P*=.04), which supported hypothesis H4 [23]. The seventh, eighth, and ninth hypotheses (ie, H7, H8, and H9) were developed to determine whether attitude, the SN, and PBC affected people’s intention to use a mobile learning platform. Our results showed that the effects attitude (*P*<.001), the SN (*P*<.001), and PBC (*P*<.001) on people’s intention to use a mobile learning platform were positive and significant. Therefore, H7, H8, and H9 were in line with the findings of various studies [49,50,56-58,63]. Our analysis strongly supported the proposed research model. The findings of other researchers [23,41,43-45,47-50,56-58,63] and our results have similarities.

Research studies have assessed the influence of the COVID-19 pandemic on modern technology, specifically the effects of the pandemic on technology that is used for learning and teaching. Technology is an effective tool that provides a new and viable platform for enabling the continuation of teaching and learning during lockdown [81]. Therefore, this study aimed to analyze the influence that COVID-19 has on teaching practices, by using machine learning algorithms. Our research model emphasized the effects of PF, which had an extraordinary influence on measuring the effects of COVID-19 on student and teacher groups. Furthermore, our analysis was able to assess the influence of the pandemic on mobile learning technologies that are used for teaching. Hence, our study helps with removing the identified gaps in the field and establishing a basis for future research on mobile learning and teaching practices.

### Theoretical and Practical Implications

Our analysis contributes to existing literature by exploring the primary impediments that hinder the effective use of mobile learning systems during the COVID-19 pandemic. This study provides several important practical findings with regard to the use and adoption of mobile learning systems in limited-income states, such as the UAE. For instance, previous research has only highlighted infrastructure as the main impediment to the use of e-learning systems [16-19], but in reality, various other factors also pose a challenge to mobile learning technology adoption. These impediments include specialized issues that relate to mobile learning frameworks. Such issues include changes in management, problems related to course designs, computer knowledge issues, and monetary issues. Based on the results of our study, we can provide helpful proposals to policy makers, designers, developers, and researchers. These proposals will enable them to achieve greater familiarity with the important elements of successful mobile learning system adoption.

The first proposal is that important technical resources for the continuous technical maintenance of mobile learning platforms must be provided by university administrations and technical support staff, to encourage the extensive adoption of mobile learning materials and prevent specialized issues or postponements. The second proposal is that the successful implementation of mobile learning technologies by students and instructors should only occur if the essential hardware, software, and internet connectivity are provided by university administrations. Additionally, these university administrations should provide consistent upgrades for technological resources. The third proposal is that designers and developers need to develop mobile learning systems that are user-friendly, easy to use, and not complicated. When students and instructors find that mobile learning systems are easy to use and user-friendly, they will be encouraged to use mobile learning systems. The fourth proposal is that policy makers at UAE universities should resort to new policies and guidelines that encourage the use of mobile learning systems among students and teachers. In addition, policy makers should adjust educational policies to guarantee an adaptable transition from traditional learning to mobile learning. Support from top management is imperative in technology progression. Moreover, technology progression requires training programs to ensure that mobile learning system–related institutional principles are being promoted and strictly followed by teachers. The fifth proposal is that the outcomes of our study can help university policy makers concentrate on enhancing teachers’ educational technology knowledge by arranging training programs on methods for using mobile learning systems. Such training programs are essential, since teachers’ educational technology–related knowledge and skills are likely to convince students to use mobile learning systems, which will lead to better teacher performance and improved student efficiency. The sixth proposal is that universities need to concentrate on promoting mobile learning systems through training courses that highlight the benefits of using mobile learning systems. Universities must also focus on developing students’ competency in using information technology. The main reason for this is that students’ expertise in computer studies and positive views on mobile learning systems have a favorable impact on the success of mobile learning systems. Based on the outcomes of our study, we can provide a better understanding of mobile learning systems and offer recommendations for effectively implementing mobile learning systems during the course of the COVID-19 pandemic.

### Limitations and Future Research

It is necessary to report on various key limitations of this study. First, caution needs to be taken when generalizing our results to other institutes in the UAE or other parts of the world. This is attributed to the fact that we only collected data from 7 education institutions. Additionally, participants were selected based on a convenience sampling technique. If these limitations are considered, future research can contribute to the generalization of our results. Second, this study only evaluated students’ actual use of mobile learning systems. Future research should also focus on teachers’ actual use of mobile learning systems, so that more information on influencing factors and system implementation can be determined.

### Recommendations

With regard to web-based teaching, a mobile learning platform is considered to be a safe environment. During the COVID-19 pandemic, web-based teaching systems have been recommended. During the lockdown, web-based teaching systems have been considered a temporary solution. The availability of machine learning has promptly provided students and teachers with self-sensing security and communication tools. For example, in the UAE, Sharjah City was affected by the spread of the SARS-CoV-2 virus, and as a result, a web-based mobile learning tool has proved to be quite useful. This mobile learning platform has various advantages over other communication platforms. First, this platform can be used on laptops and smartphones; the students of the University of Sharjah have joined and participated in classes by using this platform on their smartphones. Second, the links to each class period can be used at various times, thereby allowing students to communicate with teachers at any point in time during the day. Third, the students have been much more confident, and their feelings of fear have been minimized.

### Conclusion

This study’s results are similar to those presented in earlier research studies on the importance of variables in the TAM and TPB model [41,42,44,45]. We observed that during the COVID-19 pandemic, students were much more accepting of technology if mobile learning technology was the only available tool for learning. Our PU-related and PEOU-related results are also similar to those of other studies that have assessed the influence of PU and the PEOU on students’ acceptance of mobile learning technology. Therefore, PU and the PEOU should be considered indicators of students’ willingness to use mobile learning platforms during the COVID-19 pandemic. Furthermore, PU was highly affected by the PEOU, which indicates that if a technology is easy to use, then it is also considered useful. Additionally, according to our results, there was a significant association between students’ acceptance of mobile learning technology and the subjective norm (*P*<.001).

Studies have indicated that students’ behavior within the classroom, their behavior in daily life, and their reactions to the use of mobile learning technology highly affect their acceptance of mobile learning technology. Previous research studies [45,52-54] have also stated that the SN and students’ acceptance of mobile learning technology are associated. In the UAE, students are considerably influenced by their classmates’ behaviors. This influence has increased the sense of security and comfort of students who have attended classes during the pandemic. Furthermore, students are motivated to use mobile learning technology to spend time with people who attend the same class. Additionally, there were several variables that significantly influenced the SN, other than the PEOU and PU. According to our results, instructors’ and students’ attitudes also helped to promote the use of mobile learning platforms as a learning tool during the pandemic period. If students and teachers have positive attitudes toward the use of mobile learning tools, they will perceive such tools to be useful, enjoyable, and effort free.

Our findings are consistent with those of previous studies [82]. For example, it has been stated that peers, students, and instructors provide useful feedback that affects students’ attitudes and perceptions toward technology effectiveness. Due to the COVID-19 pandemic, fear has been on the rise. This should be considered an essential topic for future research, as the human population continues to be severely affected by the COVID-19 pandemic. The SARS-CoV-2 virus has a high probability of transmission, which is why there is a need for complete lockdown and stay-at-home strategies throughout the world [83]. In this study, we developed a model that is useful for conducting future studies, as our model can help with assessing the influence of COVID-19 during the pandemic period. Based on our study results and the rise of fear during the pandemic period, we believe that mobile learning technologies are important and useful tools that help to reduce students’ and instructors’ fear. In our study, PF highly affected PU and the PEOU. Furthermore, according to the responses we received, fear was quite evident during the pandemic period. However, mobile learning platforms maintained a high degree of PU and PEOU, which reduced fear and encouraged students to participate in their scheduled classes.

## Abbreviations

LWL: Locally Weighted Learning
PBC: perceived behavioral control
PEOU: perceived ease of use
PF: perceived fear
PLS-SEM: partial least squares-structural equation modeling
PU: perceived usefulness
SN: subjective norm
TAM: technology acceptance model
TPB: theory of planned behavior
UAE: United Arab Emirates
